# Protecting emotional wellbeing during childbirth: exploring the role of organisational regulatory processes in promoting compassion

**DOI:** 10.3389/fgwh.2025.1569334

**Published:** 2025-03-12

**Authors:** Caroline A. B. Redhead

**Affiliations:** Faculty of Business and Law, Manchester Law School, Manchester Metropolitan University, Manchester, United Kingdom

**Keywords:** therapeutic jurisprudence, compassion, maternity services, emotional wellbeing, organisational regulatory processes, organizational hierarchy, NHS constitution

## Abstract

In this article I consider how legal processes have power to facilitate or impede emotional safety and wellbeing for women and birthing people. I suggest that the use of therapeutic jurisprudence to re-view NHS Foundation Trusts’ organisational and regulatory processes can offer new insights. Therapeutic jurisprudence is an approach which pays purposeful attention to the therapeutic (or harmful) consequences of legal processes and how they impact the psychological well-being of those upon whom they act. The report of the Inquiry into maternity and neonatal services at East Kent Hospitals University NHS Foundation Trust was the catalyst for the theoretical suggestions I make in this article. In its response to this report, the Government has acknowledged the importance of a culture of honesty, compassion and safety. However, none of the Government's recommendations considers the impact of organisational regulatory processes on the provision of compassionate care. My argument here is that such processes are neither inert nor benign. Critical socio-legal literature provides clear evidence of the anti-therapeutic potential of hierarchical organisational structures, and this is confirmed by the findings of the East Kent Report. Presenting a brief, therapeutic jurisprudence-informed review of some of the findings of the East Kent report, I suggest that a re-view of NHS Trusts’ constitution and governance processes might offer the new means of tackling maternity service failures for which Bill Kirkup called in the East Kent Report, with the ultimate aim of ensuring emotional safety and wellbeing for pregnant and birthing people in childbirth.

## 1 Introduction

In this article I consider how legal processes, specifically organisational regulatory processes, have power as social attributes to facilitate or impede emotional safety and wellbeing for women and birthing people during childbirth. I suggest that the use of therapeutic jurisprudence (TJ) as a lens through which to re-view NHS Foundation Trusts’ organisational processes can offer new insights into how emotional wellbeing can be preserved and enhanced for maternity service users. TJ is an interdisciplinary school of theory and practice designed to produce scholarship which supports law reform (1). It is an approach which pays purposeful attention to the therapeutic (or harmful) consequences of legal processes (2) and how they impact the psychological well-being of those upon whom they act (3).

The report of the Inquiry into maternity and neonatal services at East Kent Hospitals University NHS Foundation Trust (the East Kent Report) (4) was the catalyst for the theoretical suggestions I make below and sits at the centre of the discussion and analysis in the article. The East Kent Report was ’somewhat different to the usual when it [came] to recommendations’ [(4), v] in that, rather than suggesting detailed changes of policy in specific areas of practice or management, it identified values-based areas for action to improve staff and patient wellbeing: giving care with compassion and kindness, teamworking with a common purpose, and responding to challenge with honesty (4). In an open letter (published as a foreword to the East Kent Report), Bill Kirkup, who led the Inquiry, noted that,

since the report of the Morecambe Bay Investigation in 2015, maternity services have been the subject of more significant policy initiatives than any other service. Yet, since then, there have been major service failures in Shrewsbury and Telford, in East Kent, and (it seems) in Nottingham. *If we do not begin to tackle this differently, there will be more* [(4), p. v, emphasis added]

I am particularly interested that, among other things, the East Kent Report linked the values-based failures which had been identified to the regulatory and governance framework, suggesting that, in failing to identify shortcomings and encourage clarity, *regulatory processes* were partially responsible for service failings and, thus, *for the harms to patients which sat at the core of the East Kent Report*. [see (4) Chapter 5, my emphasis]. It is specifically the link between organisational regulatory processes and failures of compassion that I interrogate here. I understand ‘regulatory processes’ as being broadly drawn, to include governance and decision-making practices, and organisational engagement in the interpretation and application of laws, regulation and policy. I am interested in how law manifests itself in the failures of compassion and kindness described in the East Kent Report. My contention, noting the strong correlation between staff support and patient safety [ (5), Principle 3], is that emotional safety and wellbeing for women and birthing people during childbirth might better be protected if, acting as agents to promote positive behavioural change, organisational regulatory processes *themselves* promoted compassion.

Investigation into the safety of maternity services in the UK has focused on exploration of underlying problems, such as recurring causes of perinatal mortality (6) or poor communication (7). Law/legal processes have featured, but usually in an acknowledgement of the complexity of the regulatory environment (see, for instance, the East Kent Report, para 1.50). The Government response to the East Kent Report (8) touched on leadership and management (see Recommendation 4) but did not problematise law itself as a potential variable. Thompson and colleagues (9), investigating how to ensure safe routine maternity care, went further in this direction, concluding, among other things, that the *interpretation and application* of law, regulation and governance are key factors (emphasis added). However, no previous investigation, so far as I know, has considered whether a fundamentally different conceptual and theoretical approach to *designing* regulatory processes might better support emotional safety and wellbeing for women and birthing people during childbirth. The contribution I make in this article is to sketch out the theoretical underpinnings of such an approach, linking it specifically to the constitution of NHS Foundation Trusts (10) and to the principles and values set out in the NHS Constitution (8), which underpin the NHS as a whole.

I start by providing a brief summary of the East Kent Report, identifying five key themes for my purposes in this article. I next offer a ‘wide angled’ introduction to therapeutic jurisprudence, emphasising the work it can do to identify ‘the relationship between legal arrangements and therapeutic outcomes’ [(11), p.27]. In the next section, I draw on critical corporate law scholarship to support an argument that the ‘corporate’ nature of the model constitution for NHS Foundation Trusts (10) is, in part, responsible for the gap between the NHS values in theory and in practice. Having described the background and context for my suggestions, I move in the discussion section, to argue that a therapeutic jurisprudence-informed, values-based approach to organisational regulatory processes could (re)‘operationalise’ the principles and values described in the NHS Constitution in Trusts’ organisational processes and practices. Starting from the findings of the East Kent Report, I consider how bringing TJ into the design of organisational regulatory processes might support compassionate care, with the larger aim of facilitating an organisational culture more conducive to the protection of the emotional safety and wellbeing of women and birthing people during childbirth. I conclude by proposing a qualitative, consensus-building socio-legal enquiry to interrogate the link between organisational regulatory processes and failures of compassion in maternity services. Such a project might identify and discuss participants’ views and experience of where regulatory processes can cause friction in maternity services. An empirical bioethics methodology (12) would enable integration of empirical findings with normative, TJ-informed suggestions for changes to NHS Foundation Trust governance.

## 2 The East Kent Report

All women, birthing people and their families expect that they and their baby will be cared for safely and, where tragedies happen, that they will be well supported and treated with compassion. Over the last decade, a series of investigations into failures of NHS maternity services has made visible thousands of circumstances where this has not been the case (4, 13, 14). At the time of writing, Donna Ockenden's review into the quality and safety of maternity services at Nottingham University Hospitals NHS Trust is ongoing (15). The scope of this review, once again, includes management, governance and organisational culture (16).

The panel investigating failures of care at East Kent Hospitals University NHS Foundation Trust, found that failures of teamworking, professionalism, compassion and listening were the origins of the harms identified (4). Some of those harms, attributed to the Trust's regulatory and governance processes, are the focus of this article. In the Table 1 below, I identify five themes of particular relevance to the TJ approach I discuss here.

**Table 1:** Harms attributed to the East Kent trust's regulatory and governance processes—themes for a TJ analysis.

Theme (definition)	Example [East Kent Report para number(s)]
Statistical outcomes as the only measure of birthing person/baby wellbeing (*targets*)	Use of statistics (indicating that the majority of births ended with no damage to the birthing person or the baby) to obfuscate/ignore the scale of the problem (1.11–1.13; 5.14)
Hierarchal structures disempower frontline staff (*hierarchies)*	Executive as a ‘*threatening*’ presence (4.202) A concerning divide between senior management and frontline staff (5.29)
Intimidating and coercive behaviour cannot support compassionate care for women/birthing people (*bullying*)	*I am ashamed to say I feel intimidated at work…I feel completely unsupported by our most senior staff. At times I dread going to work* (para 5.45). And see 5.47.
Workplace fosters a ‘blame’ mindset instead of reflective practices (*blame*)	*Staff are [not] supported by senior management…there is a culture of blame and recrimination* (4.154) *The consultant stormed onto the ward…and demanded to know what I had done to produce [the bad outcome]* (4.155).
Adversarial legal processes erode kind, collaborative, team-based patient care (*adversarial*)	With employment issues, even where patient safety is threatened, ‘*external support for Trusts is often unhelpful, while defence organisations mobilise their full resources*’ (1.444)

These indicative themes, with their roots in failures of organisational regulatory processes, underpin the TJ-inspired analysis which follows.

## 3 Therapeutic jurisprudence

Therapeutic jurisprudence is a legal philosophy which concerns itself with the human effects of law (17). The broad aim of TJ scholarship is to explore ways to implement law as a restorative, remedial and healing instrument, with a view to reducing its potentially harmful, emotional, psychological, relational and economic effects (18). Developed from mental health law in 1970s America (19, 20), TJ is a movement which seeks to establish more humane and psychologically optimal approaches to law and legal issues, with an emphasis on relationality and collaboration (21). The broad aims of TJ scholarship are to use the law to empower and to promote wellbeing (22) without supporting paternalism or coercion (1, 11). TJ asserts that, while they should not trump justice or other relevant legal considerations (1, 11), therapeutic effects are desirable and should generally be an aim of the law, whereas non-therapeutic effects of laws should be avoided or minimised (1). TJ is grounded in dignity (23), and founded upon, the psychology of compassion, understood as a sensitivity to, and a concern for, the suffering of [any person on whom the law acts] and a commitment to alleviating or preventing it’ [(24), 107].

TJ has been applied in a number of legal fields [for discussion, see (25)] and there is a variety of theoretical and empirical examples of its use in studies concerning health law and policy in the US (see, for example (2, 3, 26–28). TJ has not to date, however, been explored in the NHS context. This might be because the solidaristic, relational philosophy underpinning the NHS (29–31) and the values-based approach to NHS provision (8) make it a less obvious candidate for a TJ-informed analysis than the consumer/markets-based system within which healthcare services are provided in the US [see (26)]. For instance, Cerminara (26) uses TJ to situate an argument that the patient should be at the heart of US healthcare services, something which is a core principle of NHS provision (8). However, ‘market-style’ reforms have, among other things, changed the organisational context within which NHS healthcare professionals work [(32), p17]. It is this which (particularly in light of the findings of the East Kent Report) suggests that there is a gap between NHS values in theory and their application in organisational practice. My suggestion is that a TJ-inspired analysis of Trusts’ organisational processes might help close this gap. I use the five (only thinly developed) ‘East Kent themes’ to illustrate how, while organisational processes can themselves be harmful, they might equally be therapeutic (and better promote compassion). To develop this argument, I turn next to the critical corporate law literature, where similar harms have also been identified. Conscious of the limited space for discussion, I focus specifically here on hierarchical organisational structures.

## 4 Interrogating the ‘corporate’ trust

Critical corporate law scholars have recognised the damaging effects of the hierarchical ‘corporate’ model, embedded in an individualistic, profit-seeking philosophy, on the relationships and interdependencies within large corporations (33). In a company, a board of directors exercises all the powers of the company (34). The board's overall aim is (financially) to benefit the company's shareholder members (35). Directors are generally not accountable to a company's less powerful stakeholders (35, 36), including employees, ‘human resources’ whose interests and wellbeing, it has been argued, are not always respected (36). Lower-level employees might be objectified (37) and denied a voice, leading to the creation of ‘a less beneficent culture’ [(38), p154]. David Yamada has suggested that, ‘the dominance of the markets and management framework has caused many workers to surrender their personhood, at least on the job’ [(39) p527]. While not profit-making in the same way as commercial organisations, Trusts are similarly hierarchical in structure, and also driven by market-based targets and output measures (see *targets and hierarchies* themes from East Kent Report findings).

The model constitution for NHS Foundation Trusts [see (10) (the Trust Constitution)] describes an organisational structure closely related to the standard company model. A Trust is run by a board of directors, which is overseen by a council of governors tasked with holding the non-executive directors to account for the performance of the board and to represent the interests of members of the Trust and the wider public (see Trust Constitution). The East Kent Report provides clear evidence that, in that Trust at that time, a ‘less beneficent culture’ existed (see *hierarchies; bullying; blame*). There was ‘a clear disconnect between ward and Board’ (para 5.33) (*targets*; *adversarial*). We can suggest, then, that constitutional structure is relevant to the failures of organisational processes identified in the East Kent Report and, thus, that a flattening of Trusts’ corporate hierarchy could improve organisational culture and, in turn, the emotional safety and wellbeing of women and birthing people. In the discussion which follows, I use the principles of TJ, and examples from TJ scholarship, to suggest what this might look like in practice.

## 5 Discussion: restoring the trust?

We have seen that TJ is underpinned by a recognition that legal processes are a powerful social force (17), producing behaviours and consequences (40) and impacting on people's wellbeing, feelings and self-esteem (41). In seeking to harness that power to minimise the potential for *harmful* behaviours and/or consequences, TJ brings a new dimension to, but does not trump, questions of justice (41). TJ emphasises human dignity, compassion (23, 25, 42) and respect (43). Each of these is supported, in the context of organisational decision-making, by processes which mandate the involvement of people from across the organisation in a meaningful way (44). It is interesting, in passing, to note that the conduct of the East Kent Inquiry, informed by a ‘families first’ principle, adopted an explicitly compassionate, respectful (trauma-informed) process [(4), Appendix B], which, in its specific attention to each of these key values reflects a TJ-style approach [for a TJ-focused discussion of a trauma-informed process see (2)].

My suggestion is that, even within the hierarchical corporate NHS Trust model, a more enlightened organisational approach should be employed, informed by the principles and values around which the NHS Constitution is arranged, particularly compassion, respect and dignity. The NHS Constitution is intended to inform the service provision of all NHS bodies [(8), Introduction] and is currently less influential than it might be in inspiring compassionate organisational practice (31). Second, the model Trust Constitution already anticipates (but does not mandate) the involvement of staff and patients in organisational processes (10). It is therefore already open to Trust boards to ensure that hierarchies are flattened, that stakeholders (staff at all levels *and* patients) are able to engage directly with decision-makers, and to ensure that senior managers and board members are regularly present in the wards and hallways of the hospital. Policies and procedures, together with senior decision-maker education, can move to operationalise a flatter, more compassionate, organisation for the benefit of staff and, in the context of maternity services, women and birthing people in childbirth.

Anna Kawalek’s (17) TJ work with magistrates in problem-solving courts offers food for thought in this respect. Kawalek investigated the therapeutic quality of magistrates’ behavioural interactions with people appearing before them in court. The TJ values from her study (harnessing therapeutic support; engaging therapeutic dialogue; inspiring therapeutic change) speak directly to the findings of the East Kent Report as regards hierarchical behaviours, bullying and the blame culture described. Translating Kawalek's TJ values into Trusts’ organisational practices might see policies reflecting the importance of top-down therapeutic support (e.g., active engagement with team members’/patients’ views), engaging therapeutic dialogue (openly sharing information, encouraging participation, treating team members/patients/families as equals) and inspiring therapeutic change (helping team members to develop, fitting birthing processes around patients’ wishes to the extent possible). Organisational policies and procedures might be co-created, for example, at routine patient/staff board engagement meetings, or stakeholder ‘juries’ convened to discuss organisational strategy (e.g., working within guidelines), using inclusive language to maximise effectiveness and ensure wide understanding and engagement.

In terms of *adversarial* (legal) practices, David Yamada's (39) TJ-inspired work in America emphasises the importance of educating lawyers to combine legal expertise with a problem-solving approach to adversarial processes. Hospital legal/governance teams might be encouraged to do the same, to apply a preventive approach, to ask what measures might reduce adversarial practices and to prepare employee handbooks and manage people accordingly. Further, legal advisers to the board might emphasise (as per the NHS Constitution) the importance of collaborative, compassionate management, supportive relationships, staff/patient engagement (39, 45) and mediation (41). These approaches would acknowledge the importance of *relationship*s in healthcare practice, and, as colleagues and I have argued elsewhere, could better support the provision of compassionate care (46), for the benefit of relationships across a Trust's wider community (47, 48).

## 6 Conclusion

In its response to the East Kent Report (8) the Government has acknowledged the importance of a culture of honesty, compassion and safety [see NHS England (49)]. However, none of the proposed measures addresses the impact of organisational regulatory processes on the provision of compassionate care. My argument here is that such processes are neither inert nor benign. Critical socio-legal literature provides clear evidence of their anti-therapeutic potential, and this is confirmed by the findings of the East Kent Report. The organisational processes embedded in Trusts’ constitutional structure appear, thus, to be out of step with the relational, values-based underpinnings of the NHS as an organisation. My brief TJ-focused review of some of the findings of the East Kent Report suggests that a re-view of Trusts’ constitution and governance processes might offer the new means of tackling maternity service failures for which Bill Kirkup called in the East Kent Report. I suggest that empirical research involving midwives, obstetricians and pregnant and birthing people is required to explore in more depth the points of tension in everyday organisational maternity processes and practices, with the ultimate aim of ensuring emotional safety and wellbeing for pregnant and birthing people in childbirth.
